# Body image in women with HIV: a cross-sectional evaluation

**DOI:** 10.1186/1742-6405-3-17

**Published:** 2006-07-06

**Authors:** Jeannie S Huang, Shawn Harrity, Daniel Lee, Karen Becerra, Rosanne Santos, W Christopher Mathews

**Affiliations:** 1 Department of Pediatrics, University of California, San Diego, California, USA; 2 Children's Hospital, San Diego, California, USA; 3 Department of Medicine, University of California, San Diego, California, USA

## Abstract

**Background:**

HIV lipodystrophy syndrome is a recognized complication of potent antiretroviral therapy and is characterized by often dramatic changes in various body fat stores, both central and peripheral. Given prior findings of heightened body image dysphoria among HIV-infected men with lipodystrophy as compared to HIV-infected men without lipodystrophy, we sought to determine body image among HIV-infected and HIV-negative women and to determine the relationship of HIV and lipodystrophy with body image. Our a priori hypothesis was that women with HIV and lipodystrophy would have significantly poorer body image as compared to women without HIV and to women with HIV without lipodystrophy.

**Results:**

116 women responded to two previously validated self-report instruments (Body Image Quality of Life Index (BIQLI) and the Situational Inventory of Body-Image Dysphoria – Short Form (SIBID-S)) on body image. 62 (53% subjects) HIV-infected women were recruited at the university-based HIV clinic. 54 (47% subjects) HIV-negative female controls were recruited from another study evaluating bone density in otherwise healthy controls. 96% identified their sexual orientation as women having sex with men. Among the HIV-infected group, 36 reported the presence of lipodystrophic characteristics and 26 reported no lipodystrophic changes. Agreement regarding the presence of lipodystrophy between physician and subject was 0.67 as measured by the kappa coefficient of agreement. Compared to HIV-negative women, HIV-positive women demonstrated poor body image as measured by BIQLI (p = 0.0009). Compared with HIV-infected women who denied lipodystrophy, HIV-infected women with self-reported lipodystrophy demonstrated poor body image as measured by BIQLI (p = 0.02) and SIBID-S scales (p = 0.001).

**Conclusion:**

We demonstrate that HIV and lipodystrophy status among women is associated with poor body image. Universal efforts should be made in the HIV medical community to recognize body image issues particularly among persons affected by lipodystrophy so that appropriate intervention and support may be provided.

## Background

Although HIV infection in the current era of highly active antiretroviral therapy (HAART) has become a survivable and chronic condition, multi-drug regimens pose significant potential metabolic side effects. HAART has been associated with significant changes in body habitus in many patients. The HIV lipodystrophy syndrome encompasses two different patterns of changes in central and peripheral body fat stores, both of which are associated with specific antiretroviral agents. Increased visceral adiposity, hypertrophy of the posterior cervical fat pad ("buffalo hump") and increases in female breast fat is typically associated with agents of the protease inhibitor class [1], while subcutaneous fat atrophy of the extremities and face have been described primarily with agents in the nucleoside reverse transcriptase inhibitor class[2].

Many studies have demonstrated impaired psychosocial functioning in patients with the lipodystrophy syndrome as well as a negative impact on their quality of life and sexual behavior [3-7]. We previously reported notable differences in body image measures among HIV-infected men according to lipodystrophy status [8]. Prior research demonstrating notable gender differences in body image perception [9-11] suggests that body image studies among mixed male-female populations should control for gender confounding of results or evaluate gender groups separately. In the current study, we investigated body image among HIV-infected and HIV-negative women. Our a priori hypothesis was that women with HIV infection would have significantly worse body image dysphoria and lower body image related quality of life as compared to HIV-negative controls. Similarly, we proposed that women with HIV infection and lipodystrophy would have lower body image as compared to HIV-infected women without lipodystrophy.

## Results

### Demographics

Ninety-seven percent of approached patients agreed to participate, and one hundred and sixteen women participated in our survey study. Sixty-two women were HIV-positive. Ethnic composition of the entire cohort was 34% Hispanic, 10% black, 45% white, 8% Asian, 3% other. Demographic data are displayed in Table 1. There were no differences in age or sexual orientation according to HIV status; however, there was less Caucasian representation among the HIV-infected population as compared to HIV-negative control (p = 0.01). Among HIV-infected women, median CD4 count was higher (p = 0.02) and median HIV viral load (p = 0.003) was lower in women with self-reported lipodystrophy, as compared to HIV-positive women without lipodystrophy. AIDS status did not differ between HIV-positive groups (p = 0.48).

**Table 1:** Population Demographics

	Entire Cohort	HIV + with Lipodystrophy^Ω^	HIV+ without Lipodystrophy^Ω^	HIV-Control	p-value **
Number of persons	116	36	26	54	
Age (years)*	40 (33, 48)	43 (36, 50)	37 (31, 45)	40 (34, 48)	0.18^¥^
Race (white: non-white)	53: 63	16: 20	6: 20	31: 23	0.01^Σ^
Sexual Orientation (heterosexual: not)	111:5	33:3	26:0	52:2	0.27^Σ^
SIBID-S score*		2.3 (1.8, 2.8)	1.3 (0.5, 2.3)	1.4 (0.6, 2.4)	0.002^¥^
BIQLI score*		-0.7 (-1.2, 0.2)	0 (-0.6, 0.6)	0.7 (-0.5, 2)	0.0008^¥^

		HIV + with Lipodystrophy^Ω^	HIV + without Lipodystrophy^Ω^	p-value ^▲^	

CD4 count (cells/μL)*		626 (444, 742)	430 (234, 588)	0.02^¥^	
Log_10 _HIV viral load (copies/mL)*		2.6 (2.6, 2.8)	3.7 (2.6, 4.6)	0.003^¥^	
AIDS (Yes:No)		14:22	9:17	0.48^Σ^	
Believe that appearance reveals HIV status (%)		22%	0%	0.003^Σ^	

*Expressed as median (interquartile range).

^▲^Between HIV+ lipodystrophy vs. HIV+ non-lipodystrophy groups.

**Statistical comparisons among all 3 groups.

^Ω^As reported by the patient.

^¥^p-value by Kruskal-Wallis rank sum test.

^Σ^p-value by chi-square statistics.

### Lipodystrophy assessment results

Concordance between physician and self-determination of lipodystrophy status among HIV-infected women was substantial, as determined by the kappa coefficient of agreement (κ = 0.67).

### Body image and HIV and lipodystrophy status

HIV and self-reported lipodystrophy status were associated with body image subscales (Table 1). HIV-positive women, particularly HIV-positive women with lipodystrophy, had lower reported body image related quality of life scores compared to HIV-negative controls (p=0.0009). HIV-positive women with self-reported lipodystrophy reported higher body image dysphoria (SIBID-S scores) compared to HIV-positive women without lipodystrophy and HIV-negative women.  Compared with HIV-infected women who denied lipodystrophy, HIV-infected women with self-reported lipodystrophy demonstrated poor body image as measured by BIQLI (p=0.02) and SIBID-S scales (p=0.001).

Similarly, among HIV-positive women, physician rated lipodystrophy was significantly associated with both body image subscale scores. Of the HIV-infected women, 38 women demonstrated clinical evidence of lipodystrophy; 24 did not. HIV-positive women with clinical lipodystrophy had significantly (p = 0.009) lower BIQLI scores (median (IQR): -0.7 (-1.2, 0.04)) as compared to HIV-infected women who did not have clinical lipodystrophy (0.2 (-0.7, 0.8). HIV-positive women with clinical lipodystrophy also reported higher SIBID-S scores as compared to HIV-infected women without clinical lipodystrophy (2.3 (1.6, 2.8) vs. 1.5 (0.5, 2.3), p = 0.005).

22% of HIV-infected women with self-reported lipodystrophy as compared to 0% of HIV-infected women without lipodystrophy believed that others knew their HIV disease status based solely on their appearance (p = 0.003).

Among HIV-positive women, specific patient-reported lipodystrophy changes (i.e. fat atrophy or hypertrophy) were also associated with body image scales (Table 2). In particular, fat changes at the face, neck, breasts, abdomen, and arms were significantly associated with SIBID-S scores according to pair wise comparisons between fat change groups and no change groups. Only fat changes at the abdomen and breasts were significantly associated with BIQLI scores.

**Table 2:** Patient Reported Site-Specific Fat Changes and Body Image Scale Scores

**Body Site**	**Fat Change (n)**	**SIBID-S**	**BIQLI**
Face	Fat Hypertrophy (12)	2.7 (2.3, 2.9)**	-0.8 (-1.3, 0.5)*
	Fat Atrophy (12)	2.6 (2.0, 3.1)**	-1.0 (-1.3, -0.2)
	No Change (38)	1.7 (0.6, 2.2)	0 (-0.8, 0.4)

Neck	Fat Hypertrophy (15)	2.3 (2.1, 2.8)*	-1.0 (-1.3, 0)
	Fat Atrophy (1)	3.1**	1.7
	No Change (46)	1.9 (0.8, 2.5)	-0.2 (-0.9, 0.4)

Arm	Fat Hypertrophy (6)	2.3 (1.9, 3.0)	-0.7 (-1.2, 0.8)
	Fat Atrophy (17)	2.5 (2.1, 3.0)**	-0.8 (-1.3, 0.2)*
	No Change (39)	1.8 (0.5, 2.4)	-0.1 (-0.8, 0.6)

Breast	Fat Hypertrophy (18)	2.7 (2.0, 3.0)**	-1.0 (-1.3, -0.2)**
	Fat Atrophy (6)	2.4 (1.9, 3.0)	0.9 (-1.0, 2.0)
	No Change (38)	1.8 (0.5, 2.3)	-0.1 (-0.8, 0.4)

Abdomen	Fat Hypertrophy (39)	2.3 (1.7, 2.8)**	-0.7 (-1.2, 0)**
	No Change (23)	1.3 (0.5, 2.3)	0.2 (-0.4, 0.8)

Leg	Fat Hypertrophy (8)	2.3 (2.1, 3.2)*	-0.8 (-1.1, -0.5)*
	Fat Atrophy (24)	2.3 (1.6, 2.8)*	-0.5 (-1.2, 1.0)
	No Change (30)	1.8 (0.5, 2.4)	-0.05 (-0.9, 0.5)

Buttock	Fat Hypertrophy (8)	2.5 (1.0, 2.9)	-0.8 (-1.3, 1.9)
	Fat Atrophy (17)	2.3 (1.9, 2.7)	-0.8 (-1.2, 0.2)
	No Change (37)	1.9 (0.7, 2.4)	-0.1 (-0.8, 0.4)

Note: Body Image Scale scores reported as Median (IQR).

**Denotes significant difference between group medians (p < 0.025) as determined by Wilcoxon rank sum test comparing fat change group to "no change" control.

* p ≤ 0.05.

### Multiple linear regression analyses

Multiple linear regression analysis identified significant associations between HIV status and body changes caused by chronic disease, and poor body image scores. In particular, positive HIV status was associated with lower body image related quality of life, and presence of body changes caused by any chronic disease was associated with greater body image dysphoria. Results are displayed in Table 3.

**Table 3:** Multiple Regression Analyses on Body Image Measures and Selected Patient Characteristics in the Entire Cohort

**Dependent Variable: BIQLI, R**^2^** = 0.15, whole model p = 0.001**			
Variable	Estimate	Standard Error	p-value

Self-reported body changes due to any chronic illness (absent)	-0.29	0.15	0.06
Caucasian (not Caucasian)	-0.18	0.13	0.16
Age (by decade)	-0.006	0.01	0.64
**HIV status (negative)**	**-0.34**	**0.15**	**0.02**

**Dependent Variable: SIBID-S, R**^2^** = 0.17, whole model p = 0.0004**			

Variable	Estimate	Standard Error	p-value
**Self-reported body changes due to any chronic illness (absent)**	**0.45**	**0.11**	**<0.0001**
Caucasian (not Caucasian)	0.08	0.09	0.41
**Age (by decade)**	**0.02**	**0.01**	**0.05**
HIV status (negative)	-0.08	0.11	0.44

Note: The reference group is presented after each variable in parentheses. Higher BIQLI scores indicate higher body image quality of life; higher SIBID-S scores indicate higher body image dysphoria.

Among the HIV-positive group, controlling for ethnicity, multiple regression analyses were also performed to determine which site-specific patient-identified fat changes (as identified by prior notable associations demonstrated on pair-wise comparisons) predicted situational anxiety and affected body image quality of life. Significant associations between arm atrophy (reference = arm hypertrophy or no change, β = -0.79, p = 0.003), leg atrophy (reference = leg hypertrophy or no change, β = +0.69, p = 0.004), and breast hypertrophy (reference = breast atrophy or no change, β = -0.43, p = 0.03) and BIQLI scores were identified (whole model R^2 ^= 0.32, p = 0.008). Likewise, significant associations were found between arm atrophy (reference = arm hypertrophy or no change, β = +0.57, p = 0.009) and leg atrophy (reference = leg hypertrophy or no change, β = -0.35, p = 0.07), and SIBID-S scores (whole model R^2 ^= 0.39, p = 0.0006).

## Discussion

We performed an observational study among HIV-infected and HIV-negative women to determine whether HIV status and self-perceived lipodystrophy status affects body image and quality of life. Our findings suggest that HIV status and somatic changes interpreted as lipodystrophy are associated with body image in women.

Body image encompasses an individual's body-related self-perceptions and self-attitudes, and is linked to self esteem, interpersonal confidence, eating, exercise, and grooming behaviors, sexual experiences, and emotional stability [12,13]. Diagnosis with a chronic disease can have detrimental effects on self esteem and body image [14]. In our cohort, we demonstrate that diagnosis with HIV disease has a negative effect on body image related quality of life among women. Alterations in body appearances can similarly have significant effects on psychosocial well-being and quality of life. Evidence of negative interactions between body changes and body image has already been demonstrated in HIV-infected men with AIDS wasting [15] and with lipodystrophy [8]. In this study, we also demonstrate evidence of detrimental consequences on body image measures (greater body dysphoria and lower body image related quality of life) among HIV-infected women who perceive that they manifest lipodystrophy body changes.

Studies indicate that the typical Western society female prefers a thin body at all areas including chest, abdomen, and limbs [16-18]. In accordance, our data demonstrated that reduction in leg fat and breast size was associated with improvements in body image measures. However, regression modeling also suggested that reduction in arm fat was associated with greater body image dysphoria and lower body image related quality of life. One possible explanation might be that loss of fat in the leg region increases definition of muscles and results in an apparent "toned" appearance, while loss of fat in the arm region may lead to an atrophic appearance and suggestion of physical inactivity or lack of physical fitness. Our findings of increased desirability of fat reduction at the leg compared to the arm in Western culture is supported by surgery data from the United States demonstrating that the most common sites of liposuction are located at the leg (hip, thigh, knee, and calf) [19].

Some studies have demonstrated differences in how women of color view their bodies in comparison to Caucasian women [20]. However, a recent study evaluating whether ethnicity predicted acceptance of higher body weight among 801 women (24% Asian, 47% Hispanic, 16% black, 13% white) from a wide range of ages, body weights, and educational backgrounds only found lower body dissatisfaction among Asian women as compared to women of other ethnicities [21], verifying prior investigators' findings [22-24]. Of note, there were no demonstrated differences in body size ideals among Black, Hispanic, and white, non-Hispanic women [21]. Our analyses similarly did not identify ethnicity (categorized as white vs. non-white) as a modifier of reported results.

In our cohort, HIV-infected women with lipodystrophy were more likely to feel that their HIV status was discernible by their body changes as compared to HIV-infected women without lipodystrophy. We and others have reported similar findings among other HIV-infected populations [8,25]. Despite its increasing prevalence, HIV disease remains a social stigma [26-30]. The fear of HIV status discovery as revealed by overt body changes accompanied by the shame and fear of discrimination associated with HIV disease [31-33]may thus explain the heightened body image dysphoria and lower quality of life among women with HIV lipodystrophy.

Medication effects on the body and body image are often listed as reasons for non-adherence among consumers, and particularly female consumers [34]. This is similarly true for HIV-infected patients, where lipodystrophy has been demonstrated to have a detrimental impact on medical compliance with antiretroviral therapy [35-37]. In order to provide early intervention and perhaps improved outcome, routine body image screening procedures should be instituted to provide the clinician with a standardized method to better understand the full spectrum of body image concerns that patients experience and to increase and improve communication between physician and patient. In the setting of HIV infection, our findings suggest that body image concerns should be addressed and discussed between physician and patient when selecting initial antiretroviral regimens.

Interpretation of our results is subject to a number of limitations. First, this was a cross-sectional evaluation. As a result, we were not able to compare how lipodystrophy directly affects body image by assessing body image before and after somatic changes. Second, what is referred to as lipodystrophy syndrome is not a homogeneous entity and varies in severity. Nevertheless, lipodystrophy status in our study was defined both by subject and physician with good concordance, and regardless of the method of definition, HIV-infected women with lipodystrophic changes were found to have significant heightening of their situational anxiety regarding their body and reduction in their body image related quality of life as compared to HIV-infected women without lipodystrophic changes. However, by ascertaining lipodystrophy presence in a binary fashion (presence or absence) or in a directional fashion (hypertrophy, atrophy, and no change), the relationship between degrees of lipodystrophy and magnitude of body image change could not be evaluated. Fourth, there remains a significant portion of variability not explained by our current model. Further study is therefore needed to better assess the relationship between HIV status, lipodystrophy status, and body image and the contribution of HIV and lipodystrophy to the deterioration of body image among women.

## Conclusion

In conclusion, notable body image dysphoria and situational anxiety is demonstrated in women with HIV disease and particularly among HIV-infected women with lipodystrophy. The detrimental effects of antiretroviral therapy-associated lipodystrophy on body image may contribute to non-adherence among HIV-infected female consumers. Universal efforts by HIV medical caregivers should be pursued to increase recognition of body image issues in the HIV-infected population so that timely and appropriate intervention and support may be provided.

## Methods

### Participants and setting

The study protocol was approved by the University of California, San Diego Human Research Protections Program prior to study performance. HIV-infected subjects were recruited from an academic multidisciplinary adult HIV clinic and via HIV community centers in San Diego. Controls were recruited via another study evaluating bone density in women. Informed consent was obtained prior to any and all study procedures.

### Questionnaires

The Body Image Quality of Life Inventory (BIQLI) is a clinical survey instrument used to assess how an individual's body image impacts his or her life. The BIQLI uses a 7-point response format ranging from very negative (-3) to very positive (+3) effects of body image on 19 life domains [12,38]. The nineteen-item BIQLI is internally consistent and has been demonstrated to converge significantly with multiple measures of body-image evaluation as well as with body mass. The BIQLI is valuable for quantifying how persons' body image experiences affect a broad range of life domains, including sense of self, social functioning, sexuality, emotional well-being, eating, exercise, grooming, etc. The BIQLI is scored as an average numeric score of the 19 items where a more negative score reflects a more negative body image.

The Situational Inventory of Body-Image Dysphoria (SIBID) is an assessment of the frequency of negative body-image emotions across specific situational contexts. This inventory asks respondents how often they experience body-image dysphoria or distress (according to a numeric range of 0 (never) to 4 (always)) in each of 48 identified situations in both social and nonsocial contexts related to such activities as exercising, grooming, eating, intimacy, physical self-focus, and appearance alterations. Research has confirmed that this is an internally consistent, stable, and convergently valid measure of body-image affect that is responsive to body-image therapy [39-42]. Recently, a 20-item short form of the SIBID has been validated and found to correlate highly (r > .95) with the original longer version [43]. The short form of the SIBID (SIBID-S) was used in this study. The SIBID-S is scored as the numeric mean score of its 20 items where a higher score is associated with increased body image dysphoria.

In addition to the above questionnaires, subjects were asked to identify the location of their body changes, and to state whether others noticed their body changes and whether they felt that their disease status was revealed to strangers based on their body changes.

### Study procedures

After study procedures were reviewed and informed consent was obtained, subjects were asked to answer the self-administered questionnaires. A physical examination was also performed by one of two study physicians to determine presence or absence of lipodystrophy (with good inter-physician concordance demonstrated in our prior study [8]). Subjects were also asked to categorize themselves as having lipodystrophy or not and to identify where fat atrophy or hypertrophy had occurred. Among HIV-infected subjects, additional data including most recent CD4 count and HIV viral load were also obtained from the medical record.

### Response coding

Racial response categories included: white, black, Hispanic, Asian, or other; for the purposes of regression analysis, these groups were collapsed according to white or non-white origin. Self-reported lipodystrophy status and clinician-determined lipodystrophy status were coded as present or absent. HIV disease status was coded as present or absent. AIDS status was coded as meeting AIDS diagnostic criteria or not. Sexual orientation was coded as women having sex with men (heterosexual) or other. For regression analyses, age in years was represented by decade of life and CD4 count was coded per 100 cells/μL. HIV plasma viral load was log_10_-transformed prior to analysis, and specimens reported as "undetectable viral load" were coded as 400 copies/mL. Patient reported body site changes were coded as no change, fat hypertrophy, or fat atrophy. Whether others noticed a given subject's body changes and whether the subject believed that their disease status was revealed to strangers based on their body changes were coded as yes or no. BIQLI and SIBID-S scores were not modified.

### Statistical analysis

Patient groups were compared according to selected measures using chi-square statistics for categorical variables and the Kruskal-Wallis rank sum test for continuous variables. Multivariate modeling of BIQLI and SIBID-S scores was performed entering HIV and lipodystrophy status as covariates in order to determine the association between HIV and lipodystrophy status and body image scale scores. Body site specific comparisons of mean SIBID-S and BIQLI scores according to patient reported change (no change, fat hypertrophy, and fat atrophy) were performed using Wilcoxon rank sum pair wise comparisons between fat change groups and the "no change" group using p-value < 0.025 as the significance level applying the Bonferroni correction for multiple comparisons [44]. Multiple linear regression models of BIQLI and SIBID-S scores were subsequently performed among the entire cohort (entering demographic variables) and among HIV-infected women only (entering site specific variables with potential associations, defined as p ≤ 0.05). Statistical analyses were performed using JMP 5.0 (SAS Institute, Inc., Cary, NC).

## Competing interests

The author(s) declare that they have no competing interests.

## Authors' contributions

JSH conceived of the study, participated in its design and performance, and drafted the manuscript. KB and RS participated in questionnaire performance, data collection, and data analysis. WCM, SH and DL participated in subject recruitment, physician evaluations, and helped to draft the manuscript. All authors read and approved the final manuscript.
